# Uncertainty-Aware Remaining Useful Life Prediction via Synergizing TCN–Transformer Networks and Fractional Brownian Motion

**DOI:** 10.3390/e28050565

**Published:** 2026-05-18

**Authors:** Yiming Geng, Tianshuo Yu, Yan Liu, Jiayin Zhao

**Affiliations:** 1 School of Communication Engineering, Jilin University, Changchun 130022, China; 2 Key Laboratory of CNC Equipment Reliability, Ministry of Education, Changchun 130022, China; 3 School of Mechanical and Aerospace Engineering, Jilin University, Changchun 130022, China; 4 School of Mathematics, Jilin University, Changchun 130022, China

**Keywords:** fractional Brownian motion, temporal convolutional network (TCN), transformer, remaining useful life prediction, long-range dependency

## Abstract

Accurate Remaining Useful Life (RUL) prediction is pivotal for the intelligent operation and maintenance of high-precision equipment. However, existing deep learning-based prognostic methods predominantly focus on point estimations and often overlook the non-Markovian characteristics and stochastic uncertainties inherent in complex mechanical degradation. To bridge this gap, this study proposes a novel uncertainty-aware hybrid prognostic framework by synergizing TCN–Transformer architectures with fractional Brownian motion (FBM). Specifically, a TCN–Transformer hybrid network is developed to adaptively learn a multi-scale drift function, effectively capturing both localized causal features and global long-range temporal dependencies. Concurrently, the FBM component is employed to model the diffusion process, explicitly accounting for the long-range dependence and inherent stochasticity of degradation. By leveraging the first hitting time (FHT) principle, an approximate analytical expression for the RUL probability density function (PDF) is derived based on an established approximation treatment for FBM-driven degradation processes, enabling robust uncertainty quantification. Experimental results on both the XJTU-SY bearing dataset and the servo tool holder power head system dataset demonstrate that the proposed method achieves superior predictive accuracy and reliable uncertainty quantification, thereby providing effective support for condition-based maintenance and intelligent decision-making.

## 1. Introduction

Remaining useful life (RUL) prediction plays a critical role in the intelligent operation and maintenance of complex mechanical equipment, including bearings, rotating machinery, and high-precision servo systems. Reliable prognostic results can effectively reduce unexpected shutdowns, optimize maintenance scheduling, and improve system availability. However, due to nonlinear degradation evolution, stochastic disturbances, and long-range temporal dependence in practical operating environments, accurate and uncertainty-aware RUL prediction remains a challenging task. The predictive strategies are generally partitioned into three domains: mechanistic physics-based models, empirical data-driven techniques, and fusion-based hybrid strategies [1,2]. Driven by recent advancements in artificial intelligence, deep learning-based data methods have gained significant prominence, primarily due to their unparalleled efficiency in identifying hidden non-linear patterns and time-evolving features within complex monitoring datasets [3].

In industrial environments, mechanical systems are subject to various uncertainties, such as load fluctuations and sensor noise, which result in stochastic rather than deterministic degradation paths [4]. To model this randomness, stochastic processes like the Wiener process (WP) are widely used. For instance, Li et al. [5] incorporated unit variability into WPM, and Wu et al. [6] applied an adaptive nonlinear WPM for bearing analysis. Mao et al. [7] developed a Wiener-process-assisted deep incremental transfer learning method for online RUL prediction, enabling dynamic tracking of degradation trends in online monitoring data. Si et al. [8] proposed a Wiener-process-inspired semi-stochastic filtering approach for prognostics, which addresses the problem of selecting or determining the conditional distribution of condition monitoring (CM) measurements conditioned on the remaining useful life (RUL). However, standard Wiener models rely heavily on the Markov assumption, implying that future degradation depends solely on the current state, ignoring historical trends. This assumption is often invalid for complex systems like bearings and turbofan engines, which exhibit significant long-range dependence [9].

Long-range dependence characterizes persistent correlations among degradation states separated by long time intervals, reflecting the non-Markovian nature of degradation dynamics. Fractional Brownian motion (FBM) has therefore been introduced to represent such memory effects in degradation modeling. Xi et al. [10] developed an FBM-driven degradation model and applied Monte Carlo simulation techniques to estimate system RUL, whereas Zhang et al. [11] derived an approximate probability density function for FBM-based degradation by leveraging weak convergence theory to obtain analytical RUL solutions. These studies indicate that FBM-based models are more consistent with degradation mechanisms observed in practical equipment. However, FBM-oriented approaches often encounter difficulties in parameter estimation and generally yield lower prediction accuracy when compared with contemporary deep learning-based methods.

Driven by the rapid development of the Industrial Internet of Things (IIoT), degradation signals collected from industrial assets have increasingly evolved into voluminous and high-dimensional datasets. Advanced deep learning frameworks, notably long short-term memory (LSTM) units, convolutional neural networks (CNNs), and Transformer architectures, demonstrate powerful representation-learning potential and have proven highly effective in RUL forecasting tasks [12]. For instance, a hybrid structure coupling LSTM with attention modules was developed by Chen et al. [13] to synthesize handcrafted attributes with automatically extracted features, while a self-evolving cascade LSTM architecture was introduced by Hu et al. [14] to achieve high-precision life estimates for turbofan systems. Although LSTM networks alleviate gradient explosion and vanishing issues inherent in traditional recurrent neural networks, they may still suffer from information loss and limited exploitation of long-term dependencies when handling extended time-series data [15]. To mitigate these limitations, Transformer-based architectures have been introduced for RUL prediction [16] due to their capability to model global temporal dependencies via self-attention mechanisms. For instance, Zhang et al. [17] proposed an extended Transformer model for multi-sensor RUL estimation. Despite their strengths in capturing long-range correlations, Transformer models may be less effective in extracting local degradation features because of their emphasis on global dependency modeling.

Temporal convolutional networks (TCN) offer an alternative modeling paradigm by utilizing dilated convolutions to efficiently capture local temporal patterns [18]. Qiu et al. [19] developed a TCN-based approach for degradation stage division and RUL prediction, while Cao et al. [20] proposed a TCN-RSA framework that integrates time–frequency features to enhance prediction accuracy. Although TCN-based models are effective in local feature extraction, they remain limited in their ability to capture long-span global dependencies. Transformer architectures are effective in capturing long-range global dependencies. In addition, Zhang et al. [21] proposed an attention-based temporal convolutional network method for aero-engine RUL prediction, which effectively improved the accuracy of life prediction. Gao et al. [22] developed a degradation-aware multiscale temporal memory Transformer framework, which effectively detected state transitions at an early stage and enhanced the accuracy of RUL prediction for industrial robots. Consequently, integrating TCN with Transformer architectures has emerged as a promising solution, enabling complementary modeling of local and global temporal characteristics through attention mechanisms and parallel feature interactions [23]. The proficiency of fused TCN–Transformer architectures in distilling multifaceted temporal signatures has been evidenced by research in vehicle path forecasting [24]. Despite these strengths, prevailing deep learning-driven RUL prognosis models are predominantly centered on single-value point estimations, offering scant functionality for the explicit quantification of prediction uncertainty.

Existing hybrid prognostic models mainly focus on improving feature extraction or nonlinear mapping accuracy, but most of them still remain within a deterministic point-estimation paradigm. In contrast, the proposed framework integrates a TCN–Transformer network with an FBM-based stochastic degradation model in a complementary manner: the deep network is used to learn the nonlinear drift term from data, while FBM is introduced to describe long-range dependence and stochastic diffusion. Therefore, the proposed method differs from existing hybrid approaches not only in network architecture, but also in its explicit probabilistic treatment of degradation uncertainty and non-Markovian dynamics. To address these identified limitations, this study introduces a contemporary uncertainty-aware RUL prognosis architecture grounded in a TCN–Transformer–FBM degradation paradigm. Within the developed scheme, the degradation evolution is partitioned into two primary elements: a drift component and a diffusion component. Specifically, a TCN–Transformer hybrid is utilized to adaptively extract localized degradation features alongside global temporal dependencies for the drift term, whereas the diffusion term leverages fractional Brownian motion to simulate inherent stochastic fluctuations. By synthesizing data-centric learning with probabilistic degradation theory, the presented methodology seeks to enhance both the precision and robustness of RUL estimates. The performance of the proposed framework is rigorously assessed using an open-source bearing dataset in conjunction with a specialized degradation dataset from a servo tool holder power head system.

The primary highlights of this research are outlined as follows:

(1) A versatile uncertainty assessment architecture for RUL forecasting is established by systematically integrating the persistent memory effects of mechanical degradation through a TCN–Transformer–FBM framework.
(2) A near-exact analytical solution for the RUL probability density function is formulated, facilitating a rigorous numerical assessment of the prognostic uncertainty associated with the introduced model.
(3) By synergistically capturing macroscopic degradation trajectories alongside microscopic random variances, the presented technique elevates the resilience and precision of life estimation across varied operational environments.

The rest of this work is partitioned into several key segments. In Section 2, the nonlinear FBM degradation modeling methodology is established, complemented by an introduction to the TCN–Transformer-driven drift mechanism. Section 3 presents the proposed RUL prediction methodology along with parameter estimation procedures. Section 4 provides experimental validation using bearing data and degradation data from the servo tool holder power head system. Section 5 concludes the paper.

## 2. Basic Theory

### 2.1. A. Nonlinear Fractional Brownian Motion

The nonlinear FBM-based degradation paradigm is constructed from two integral parts: a drift component delineating the primary degradation path and a diffusion component capturing memory-enhanced stochastic dynamics. For a system with a degradation state F(t) at time *t*, the mathematical representation is formulated as follows:

(1) $$F(t)=F(0)+\alpha \int_{0}^{t}\eta (t;\theta )dt+\sigma B_{G}(t)$$

In this model, F(0) represents the baseline degradation state. The parameter α captures the inherent variance in degradation patterns across similar units and is modeled as a normally distributed random variable, $\alpha \sim N(\mu_{\alpha },\sigma_{\alpha }^{2})$, assuming statistical independence between individual units. Furthermore, σ signifies the diffusion coefficient, while $B_{G}$ denotes the fractional Brownian motion—a continuous Gaussian process characterized by stationary increments, a zero-mean property, and a specific covariance structure.

### 2.2. B. TCN Neural Network

**Figure 1:**
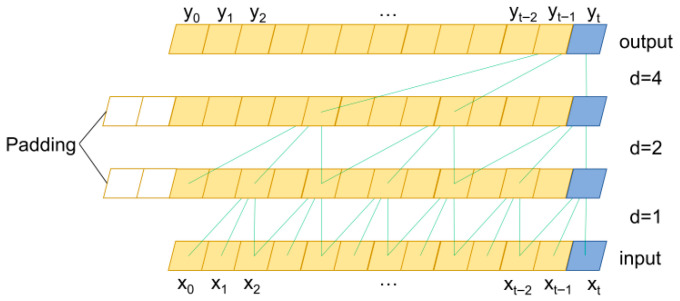
Structure of TCN.

As illustrated in Figure 1, a temporal convolutional network (TCN) featuring dilated causal convolutions is implemented to efficiently extract localized time-series dependencies while maintaining strict temporal causality. This design prevents information leakage from the future by ensuring outputs depend solely on historical data. To expand the receptive field efficiently without an excessive increase in network depth or computational cost, an exponential dilation rate *d* is applied across layers. Furthermore, residual connections are integrated within the network structure to facilitate the training of deeper layers and mitigate the vanishing gradient problem, enabling the model to fit complex nonlinear data robustly.

**Figure 2:**
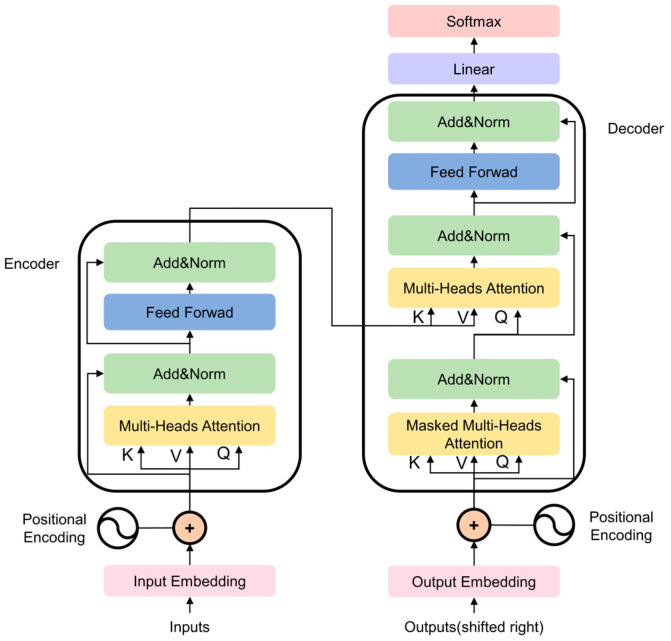
Structure of Transformer.

Contrastingly, the Transformer framework utilizes self-attention mechanisms to capture extensive global dependencies, complementing the localized focus of TCN. As illustrated in Figure 2, the architecture consists of an encoder–decoder stack; the former integrates multi-head self-attention with position-wise feed-forward networks via residual normalization, while the latter ensures predictive causality through masked attention layers. Here, “embedding” denotes the feature embedding used in the Transformer architecture and should not be confused with phase-space embedding in nonlinear dynamical system analysis.

**Figure 3:**
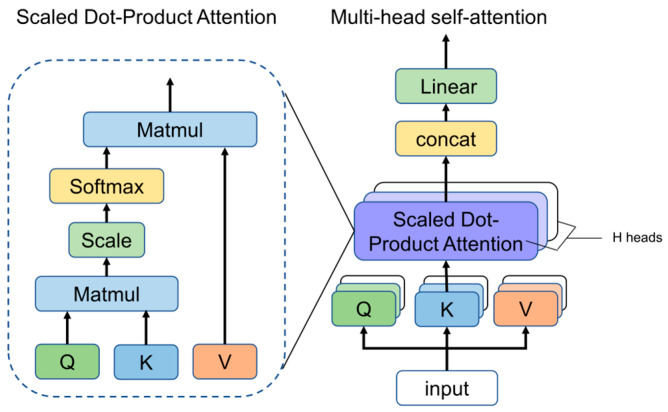
Structure of multi head attention mechanism.

To preserve sequence order, positional encodings embed temporal context into the input features. As illustrated in Figure 3, the multi-head mechanism utilizes parallel self-attention units to compute attention weights across diverse subspaces simultaneously. This adaptive weighting enables the model to prioritize critical temporal information across various representation levels.

## 3. Proposed Method

**Figure 4:**
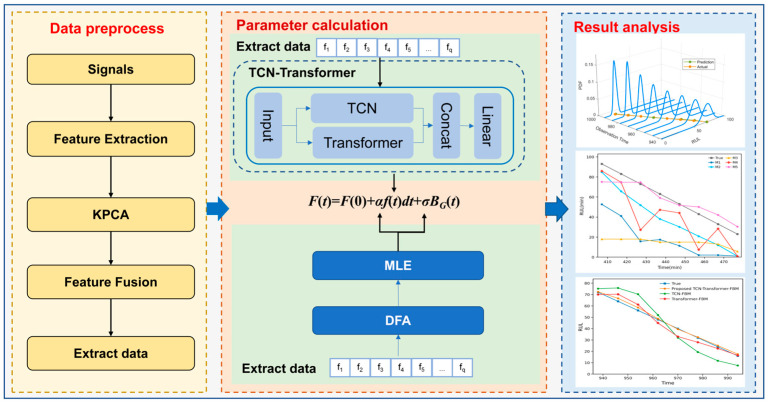
Flowchart of the proposed TCN–Transformer–FBM methodology.

As illustrated in Figure 4, the proposed TCN–Transformer–FBM framework is organized into three core phases: data preprocessing, parameter estimation, and result evaluation. Preprocessing begins by extracting time-domain features from raw signals, followed by kernel principal component analysis (KPCA) to reduce dimensionality, retaining a 95% contribution rate to construct the fused input dataset. The subsequent parameter stage involves determining model coefficients and optimizing network hyperparameters via H-index analysis, maximum likelihood estimation (MLE), and the fminsearch function in MATLAB R2023b for numerical optimization. Finally, the analysis stage validates the methodology through rigorous performance assessments and comparative studies against existing models.

### 3.1. A. The Proposed TCN–Transformer Network Architecture

**Figure 5:**
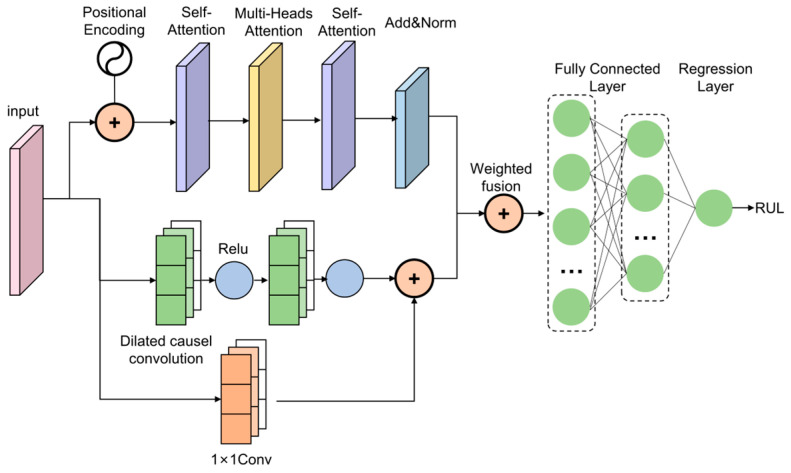
Structure of TCN–Transformer. The upper branch represents the Transformer, while the lower branch depicts the TCN. The weighted fusion of the parallel output features from the two branches is illustrated.

To proficiently identify temporal correlations within input sequences, the utilized architecture synthesizes a TCN encoder with a Transformer, leveraging weighted fusion to enhance the representation of diverse output features. A conceptual illustration of this prognostic framework is provided in Figure 5. Within this hybrid model, the application of weighted fusion boosts predictive accuracy by synergizing outputs from the constituent modules. This strategy successfully consolidates the specialized strengths of both TCN and Transformer for time-series analysis, ultimately yielding superior overall performance.

### 3.2. B. The Proposed TCN–Transformer–FBM Model

The proposed TCN–Transformer–FBM degradation model is built on a nonlinear FBM process and therefore does not satisfy the Markov property. As a result, a closed-form analytical expression for the first hitting time is generally unavailable. To obtain a tractable RUL density, the FBM-driven degradation process is approximated by a Brownian-motion-based process with an adjusted time scale under the weak convergence framework and weighted random sum treatment, following established studies on FBM-based degradation modeling [25]. This treatment preserves the main long-range dependence characteristics of FBM in an approximate sense, making it possible to derive an approximate RUL probability density function. It should be noted that this treatment is introduced for analytical tractability rather than as an exact equivalence between FBM and standard Brownian motion. In the proposed framework, the degradation process is decomposed into two complementary parts: a drift term and a diffusion term. The drift term describes the dominant deterministic degradation trend and is learned adaptively from historical monitoring data by the TCN–Transformer network. In contrast, the diffusion term captures stochastic perturbations and long-range dependence that are not fully represented by the deterministic drift component alone. Therefore, the proposed model combines data-driven trend learning with stochastic-process-based uncertainty modeling in a unified form.

Assuming an initial state F(0)=0 and defining Λ(t;θ) as the cumulative drift integral $\int_{0}^{t}\eta (t;\theta )dt$, the nonlinear FBM-based degradation model is reformulated as:

(2) $$F(t)=\alpha \Lambda (t;\theta )+\sigma B_{G}(t)$$

By applying the First Hitting Time (FHT) principle with a set failure threshold ω, the RUL at observation time $t_{k}$ can be defined by the following expression:

(3) $$RUL_{k}=\inf\{l_{k}:x(t_{k}+l_{k})⩾\omega |X\}$$

Within the TCN–Transformer–FBM framework presented in this study, a TCN–Transformer neural network is utilized to characterize the drift function. By setting Λ(t;θ)=f(t) and defining the relative failure threshold as $\omega_{k}=\omega -x\left(t_{k}\right)$, while approximating the derivative $df\left(t_{k}+l_{k}\right)/dl_{k}$ using the discrete difference $\left(f\left(t_{k}+l_{k}+\Delta l\right)-f\left(t_{k}+l_{k}\right)\right)/\Delta l$, the approximate probability density function (PDF) for the prognostic model is derived as:

(4) $$f_{k}(l_{k})=\frac{g_{k}(l_{k})}{\int_{0}^{\infty }g_{k}(l_{k})dl_{k}}$$

In this context, the term $g_{k}\left(l_{k}\right)$ is calculated as:

(5) $$\begin{array}{c} g_{k}\left(l_{k}\right)=\frac{\omega_{k}u_{k}-v_{k}c_{k}}{\Delta h\left(t_{k}+l_{k}\right)\sqrt{2\pi u_{k}^{3}}}\frac{h\left(t_{k}+l_{k}+\Delta l\right)-h\left(t_{k}+l_{k}\right)}{\Delta l}\\ \times \exp\left\{-\frac{{\left[\omega_{k}-\mu_{\alpha }f\left(t_{k}+l_{k}\right)+\mu_{\alpha }f\left(t_{k}\right)\right]}^{2}}{2u_{k}}\right\} \end{array}$$

(6) $$u_{k}={\left(f\left(t_{k}+l_{k}\right)-f\left(t_{k}\right)\right)}^{2}\sigma_{\alpha }^{2}+\sigma^{2}\left(h\left(t_{k}+l_{k}\right)-h\left(t_{k}\right)\right)$$

(7) $$c_{k}=f\left(t_{k}+l_{k}\right)-f\left(t_{k}\right)-\left(f\left(t_{k}+l_{k}+\Delta l\right)-f\left(t_{k}+l_{k}\right)\right)\frac{\left(h\left(t_{k}+l_{k}\right)-h\left(t_{k}\right)\right)}{h\left(t_{k}+l_{k}+\Delta l\right)-h\left(t_{k}+l_{k}\right)}\;$$

(8) $$v_{k}=\left(f\left(t_{k}+l_{k}\right)-f\left(t_{k}\right)\right)\sigma_{\alpha }^{2}\omega_{k}+\mu_{\alpha }\sigma^{2}\left(h\left(t_{k}+l_{k}\right)-h\left(t_{k}\right)\right)$$

### 3.3. C. Parameter Estimation of Degradation Model

Use the detrended fluctuation analysis (DFA) method to estimate the Hurst exponent (G). The DFA method has strong applicability and good adaptability to nonlinear data, and is commonly used for the analysis of various complex systems and signals [26]. The basic idea of DFA is to remove the trend component of time series and analyze the fluctuation properties after removing the trend. It has strong robustness to trends and periodic changes in data, so it can more accurately capture the long-term correlation of data. The specific process is as follows:

(1) Prepare time series data.
(2) Calculate the cumulative deviation $y(t)=\sum_{i=1}^{t}\left[x\left(i\right)-\bar{x}\right]$.
(3) Equal length segmentation of the sequence yields m=[Q/s]  intervals.
(4) Detrend by polynomial or linear fitting on each interval.
(5) Calculate the mean square error for after detrending each interval $D^{2}(v,s)=\frac{1}{s}\sum_{i=1}^{s}{\left[y\left(i\right)-y_{v}(i)\right]}^{2}$.
(6) Calculate the DFA fluctuation function $D(s)={\left[\frac{1}{2m}\sum_{v=1}^{2m}D^{2}(v,s)\right]}^{\frac{1}{2}}$.
(7) Fit D(s) and s in double logarithmic coordinates to obtain the line slope G.
(8) The physical significance of the G index is categorized as follows: a value of G=0.5 signifies a memoryless random process; 0<G<0.5 indicates anti-persistence characterized by a negative correlation; and 0.5<G<1 denotes a persistent process exhibiting a positive correlation.

Assuming that there are *P* devices’ degradation data used to fit degradation parameters, denoted as $F={\left(F_{1},F_{2},\ldots ,F_{P}\right)}^{\prime }$, where each device’s degradation data contains Q terms, denoted as:

(9) $$F_{p}={\left(f_{p}\left(t_{1}\right),f_{p}\left(t_{2}\right),\ldots ,f_{p}\left(t_{Q}\right)\right)}^{\prime },p=1,2,\ldots ,P\;$$

(10) $$\Phi ={\left(\Lambda \left(t_{1};\theta \right),\Lambda \left(t_{2};\theta \right),\ldots ,\Lambda \left(t_{Q};\theta \right)\right)}^{\prime }\;$$

(11) $$B_{G}={\left(B_{G}\left(t_{1}\right),B_{G}\left(t_{2}\right),\ldots ,B_{G}\left(t_{Q}\right)\right)}^{\prime }$$

Observation model can be changed to $F_{p}=\alpha_{p}\Phi +\sigma B_{G}$, and $F_{p}$ follows a distribution $F_{p}\sim N\left(\mu_{\alpha }\Phi ,\Sigma \right)$. Where

(12) $$\Sigma =\sigma_{\alpha }^{2}\Phi \Phi^{\prime }+\Xi =\sigma_{\alpha }^{2}\Phi \Phi^{\prime }+\sigma^{2}Q$$

(13) $$Q=\left[\begin{array}{cccc} E\left(B_{G}\left(t_{1}\right)B_{G}\left(t_{1}\right)\right) & E\left(B_{G}\left(t_{1}\right)B_{G}\left(t_{2}\right)\right) & \cdots & E\left(B_{G}\left(t_{1}\right)B_{G}\left(t_{Q}\right)\right)\\ E\left(B_{G}\left(t_{1}\right)B_{G}\left(t_{2}\right)\right) & E\left(B_{G}\left(t_{2}\right)B_{G}\left(t_{2}\right)\right) & \cdots & E\left(B_{G}\left(t_{2}\right)B_{G}\left(t_{Q}\right)\right)\\ \vdots & \vdots & \ddots & \vdots \\ E\left(B_{G}\left(t_{1}\right)B_{G}\left(t_{Q}\right)\right) & E\left(B_{G}\left(t_{2}\right)B_{G}\left(t_{Q}\right)\right) & \cdots & E\left(B_{G}\left(t_{Q}\right)B_{G}\left(t_{Q}\right)\right) \end{array}\right]$$

(14) $$E\left(B_{G}\left(t_{i}\right)B_{G}\left(t_{j}\right)\right)=\frac{1}{2}\left(t_{i}^{2G}+t_{j}^{2G}-{\left|t_{i}-t_{j}\right|}^{2G}\right),i,j=1,2,\ldots ,Q$$

Moreover, the maximum likelihood function can be derived as follows:

(15) $$\begin{array}{c} l(\Omega |X)=-\frac{PQ\ln2\pi }{2}-\frac{P}{2}\ln\left|\Sigma \right|\\ -\frac{1}{2}\sum_{p=1}^{P}{\left(F_{p}-\mu_{\alpha }\Phi \right)}^{\prime }\Sigma^{-1}\left(F_{p}-\mu_{\alpha }\Phi \right) \end{array}$$

(16) $$\left|\Sigma \right|=\left|\Xi \right|\left(\sigma_{\alpha }^{2}\Phi^{\prime }\Xi^{-1}\Phi +1\right)\;$$

(17) $$\Sigma^{-1}=\Xi^{-1}-\frac{\sigma_{\alpha }^{2}}{\sigma_{\alpha }^{2}\Phi^{\prime }\Psi^{-1}\Phi +1}\Xi^{-1}\Phi \Phi^{\prime }\Xi^{-1}\;$$

By employing the partial derivative of the parameter sum, as defined by (13), the estimated values of these two parameters can be obtained as follows:

(18) $${\hat{\mu }}_{\alpha }=\frac{\sum_{p=1}^{P}\Phi^{\prime }\Xi^{-1}F_{p}}{P\Phi^{\prime }\Xi^{-1}\Phi }\;$$

(19) $${\hat{\sigma }}_{\alpha }={\left\{\frac{1}{P{\left(\Phi^{\prime }\Xi^{-1}\Phi \right)}^{2}}\sum_{p=1}^{P}{\left(F_{p}-\mu_{\alpha }\Phi \right)}^{\prime }\Xi^{-1}\Phi \Phi^{\prime }\Xi^{-1}\left(F_{p}-\mu_{\alpha }\Phi \right)-\frac{1}{\Phi^{\prime }\Xi^{-1}\Phi }\right\}}^{\frac{1}{2}}\;$$

Substitution (18) and (19) into (15) yields the conditional log-likelihood function, with $\Xi =\sigma^{2}Q$ of particular interest. When σ is sufficiently small, the result will be beyond the computable range of the computer. In such cases, the determinant multiplication property can be employed to separate the calculations. The expression is as follows:

(20) $$\begin{array}{c} l\left(\beta ,\sigma ,G|F,{\hat{\mu }}_{\alpha },{\hat{\sigma }}_{\alpha }\right)=-\frac{PQ\ln2\pi \sigma^{2}}{2}\\ -\frac{P}{2}\ln\left[\frac{\sum_{p=1}^{P}{\left(\Phi^{\prime }\Xi^{-1}F_{p}\right)}^{2}}{P\Phi^{\prime }\Xi^{-1}\Phi }-\frac{{\left(\sum_{p=1}^{P}\Phi^{\prime }\Xi^{-1}F_{p}\right)}^{2}}{P^{2}\Phi^{\prime }\Xi^{-1}\Phi }\right]\\ -\frac{P}{2}\ln\left|Q\right|-\frac{P}{2}\\ -\frac{1}{2}\left[\sum_{p=1}^{P}F_{p}^{\prime }\Xi^{-1}F_{p}-\frac{\sum_{p=1}^{P}{\left(\Phi^{\prime }\Xi^{-1}F_{p}\right)}^{2}}{\Phi^{\prime }\Xi^{-1}\Phi }\right] \end{array}$$

The complex nonlinear function on the right-hand side of (20) renders the closed form of maximum likelihood estimation difficult to obtain by taking the partial derivative of β,σ. The fminsearch function in MATLAB should therefore be used to calculate the final estimate of β,σ.

## 4. Case Study

To quantitatively evaluate the predictive performance, RMSE, MAPE, NLL, and CRPS are adopted as evaluation metrics, as defined in Equations (21)–(24).

(21) $$RMSE=\sqrt{\frac{1}{n}\sum_{i=1}^{n}{\left(y_{i}-{\hat{y}}_{i}\right)}^{2}}$$

(22) $$MAPE=\frac{1}{n}\sum_{i=1}^{n}\left|\frac{y_{i}-{\hat{y}}_{i}}{y_{i}}\right|\times 100$$

(23) $$NLL=-\frac{1}{N}\sum_{i=1}^{N}\log\left(\frac{1}{\sqrt{2\pi \sigma_{i}}}exp\left[-\frac{(y_{i}-\mu_{i}{)}^{2}}{2\sigma_{i}^{2}}\right]\right)$$

(24) $$CRPS=\frac{1}{N}\sum_{i=1}^{N}\int_{-\infty }^{+\infty }{\left(F_{i}(z)-1(z\geq y_{i})\right)}^{2}dz$$

where n represents the number of observation points, $y_{i}$ is the actual value of the life of the i-th observation point, and ${\hat{y}}_{i}$ is the predicted life value at the same point.

### 4.1. Case 1: XJTU-SY Bearing Dataset

#### 4.1.1. Bearing Dataset Case Study

**Figure 6:**
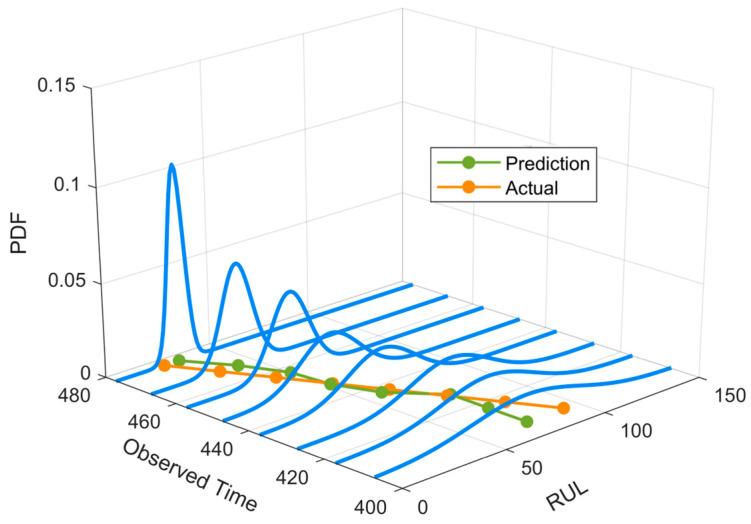
Predicted RUL probability density function (PDF) of the XJTU-SY bearing dataset based on the proposed TCN–Transformer–FBM method.

Case 1 was conducted using the vertical vibration signal of bearing 2–3 from the XJTU-SY bearing dataset, which contains a total of 533 sampling points. For the raw vibration monitoring data, time-frequency degradation features were first extracted, and KPCA was then employed for dimensionality reduction. The principal components were subsequently fused to construct the final degradation trajectory. On this basis, an adaptive point-selection strategy was used to construct the degradation dataset for model training. Specifically, for Case 1, 250 points were adaptively selected to form one sample, and a total of 20 degradation sequences were generated. The failure threshold was set to 0.7. During model training, the key hyperparameter settings of the TCN–Transformer network are listed in Table 1. According to the parameter estimation procedure described in Section 3.3, the stochastic-process parameters for this case are directly obtained as G=0.9500, $\mu_{\alpha }=0.0545$, $\sigma_{\alpha }=0.1708$, and $\sigma_{\beta }=0.0096$. The prediction results are presented in Figure 6.

As shown in Figure 6, the proposed method is able to track the overall downward trend of the bearing RUL effectively and maintains good predictive consistency across different observation points. In general, some deviation is observed at earlier observation stages; however, as the degradation process enters the middle and late stages, the agreement between the predicted and actual RUL becomes significantly better, indicating that the proposed method is more capable of capturing the accelerated degradation stage. In particular, at several critical observation points, the predicted values are already close to the actual ones, demonstrating that the model can accurately capture the key transition from slow degradation to rapid degradation. Overall, the results suggest that the proposed method can effectively learn the nonlinear trend information embedded in the degradation trajectory while stably characterizing the stochastic fluctuations during degradation, thereby achieving accurate RUL prediction.

#### 4.1.2. Ablation Study

**Figure 7:**
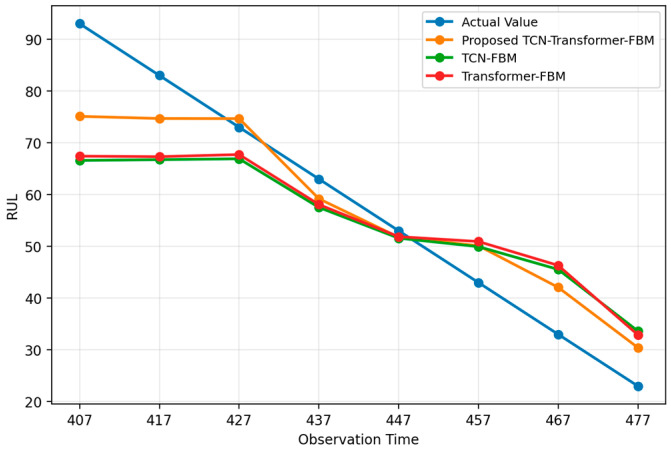
Results of the ablation study on the XJTU-SY bearing dataset.

To verify the necessity of the parallel TCN–Transformer architecture, two ablation variants, namely TCN–FBM and Transformer–FBM, were further constructed. The corresponding results are shown in Figure 7 and Table 2. As can be seen from Figure 7 and Table 2, the proposed method achieves superior overall performance in terms of RMSE, MAPE, NLL, and CRPS, indicating that the simultaneous integration of local temporal feature extraction and global dependency modeling is essential for improving bearing RUL prediction performance.

A closer comparison reveals that TCN-FBM performs better overall than Transformer–FBM, suggesting that local degradation patterns and short-term fluctuations contribute more directly to life prediction in Case 1. Nevertheless, TCN-FBM still underperforms the proposed method, indicating that relying solely on local convolutional features is insufficient to fully characterize the long-range dependencies in the degradation process. By contrast, the proposed method enhances both local sensitivity and global modeling capability through the parallel fusion of TCN and Transformer, thereby achieving more favorable results in both point prediction accuracy and probabilistic distribution characterization. In addition, the parameter comparison in Table 2 shows that the proposed method yields a lower diffusion variance, implying that the parallel fusion architecture is more effective in suppressing stochastic noise during degradation trend fitting.

#### 4.1.3. Comparative Analysis

**Figure 8:**
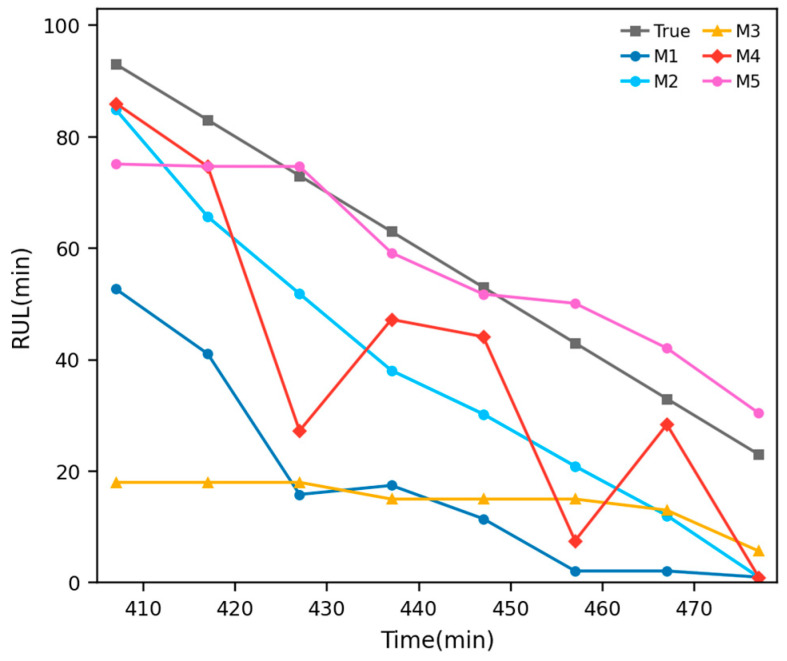
Comparative RUL prediction results on the XJTU-SY bearing dataset.

To further validate the effectiveness of the proposed method, four representative approaches were selected as benchmark methods in Case 1: M1 denotes the power function Wiener process (POW–Wiener) [27], M2 denotes the exponential function Wiener process (EXP–Wiener) [27], M3 denotes the LSTM–Wiener model [28], M4 denotes the power function fractional Brownian motion model (POW-FBM) [25], and M5 denotes the proposed TCN–Transformer–FBM method. The corresponding comparison results are shown in Table 3 and Figure 8.

As shown in Table 3 and Figure 8, M5 clearly outperforms M1–M4 overall. Specifically, M1 and M3 exhibit relatively large prediction errors, indicating that neither a Wiener process with a fixed drift form nor an LSTM–Wiener formulation alone can adequately describe the complex nonlinear characteristics of the bearing degradation process. Although M2 and M4 perform better than M1 and M3, their overall error levels remain substantially higher than those of M5, suggesting that even with an exponential drift or FBM-based memory effect, a pre-specified drift form still lacks sufficient adaptability for complex degradation trajectories. By contrast, M5 adaptively learns the degradation drift term via the TCN–Transformer architecture and further models the long-range dependence and stochastic diffusion process through FBM. As a result, it achieves the best performance in trend tracking, error control, and probabilistic prediction consistency. This demonstrates that the superiority of the proposed method lies not only in the network architecture itself, but also in the unified modeling mechanism of data-driven drift learning plus stochastic-process-based uncertainty quantification.

### 4.2. Case 2: Servo Tool Holder Power Head System Dataset

#### 4.2.1. Servo Tool Holder Case Study

**Figure 9:**
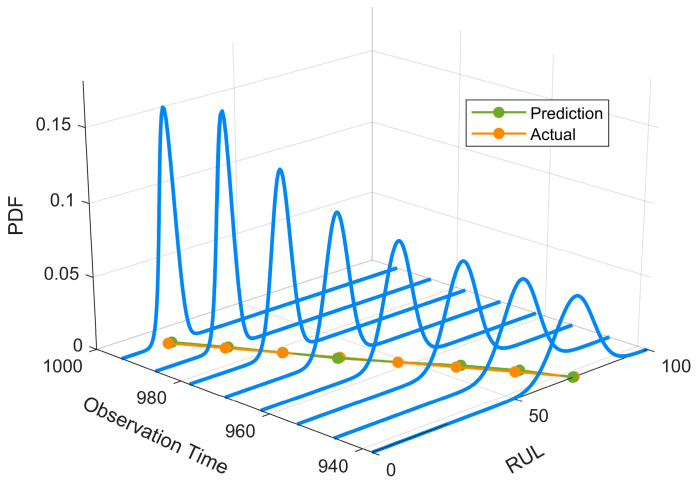
Predicted RUL probability density function (PDF) of the servo tool holder power head system dataset based on the proposed TCN–Transformer–FBM method.

Case 2 was conducted using the full-life degradation dataset of a servo tool holder power head system. The data were collected from a reliability test bench, where a constant loading condition was applied by an eddy-current dynamometer under a torque of 10 Nm and a rotational speed of 1400 rpm. Signals were sampled at 10 kHz, with a 5 s segment collected every 3 h, resulting in a total of 1028 samples. As in Case 1, time-frequency degradation features were first extracted from the raw monitoring data, and KPCA was then applied to construct the degradation trajectory. Based on this trajectory, an adaptive point-selection strategy was used to construct the degradation dataset for model training. Specifically, for Case 2, 300 points were adaptively selected to form one sample, and a total of 20 degradation sequences were generated. According to the degradation evolution of this case, the failure threshold was set to 0.9. Based on the parameter estimation procedure in Section 3.3, the corresponding stochastic-process parameters are directly obtained as G=0.9500, $\mu_{\alpha }=0.00026$, $\sigma_{\alpha }=0.0032$, and $\sigma_{\beta }=1.5000$. The predicted RUL probability density function (PDF) distributions for the tool holder power head system are visualized in Figure 9.

As shown in Figure 9, the proposed method is able to track the decreasing trend of the actual RUL more accurately in Case 2, and the predicted curve remains highly consistent with the true curve, especially in the middle and late degradation stages where high prediction stability is still maintained. Compared with Case 1, the prediction deviations at different observation points in Case 2 are generally smaller, indicating that the proposed model can still effectively extract key degradation information and achieve stable prediction on the power head system data, which exhibit stronger engineering specificity and more pronounced stage-wise degradation characteristics. The updated results further show that the proposed method not only provides higher point prediction accuracy, but also yields a more concentrated and reasonable probabilistic distribution. Overall, Case 2 further confirms the applicability and robustness of the proposed method in complex industrial degradation scenarios.

#### 4.2.2. Ablation Study

**Figure 10:**
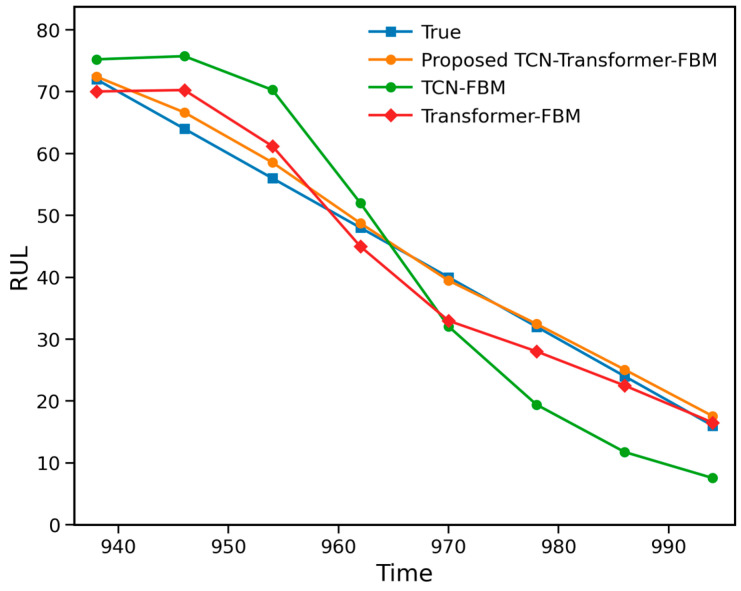
Results of the ablation study on the servo tool holder power head system dataset.

The ablation results for Case 2 are shown in Table 4 and Figure 10. It can be observed that the proposed method demonstrates a much clearer performance advantage over TCN-FBM and Transformer–FBM. The overall error of TCN-FBM is relatively large, indicating that local convolutional feature extraction alone is insufficient to fully characterize the global degradation evolution of the power head system. Transformer–FBM shows a clear improvement over TCN-FBM, suggesting that global temporal dependency modeling is more important for this industrial object; however, its overall performance still remains inferior to that of the proposed method.

A more detailed examination of Table 4 and Figure 10 shows that TCN-FBM tends to overestimate RUL at earlier observation points and significantly underestimate it at later stages, reflecting the instability of a purely local modeling strategy in complex industrial degradation scenarios. Transformer–FBM exhibits a smoother overall prediction trend yet still fails to fully match the true RUL trajectory. By contrast, the proposed method maintains better trend consistency and smaller deviations across all observation points. This indicates that the parallel fusion of TCN and Transformer indeed enhances both local degradation-detail perception and global dependency modeling capability, thereby significantly improving prediction accuracy and probabilistic prediction quality.

#### 4.2.3. Comparative Analysis and Discussion

**Figure 11:**
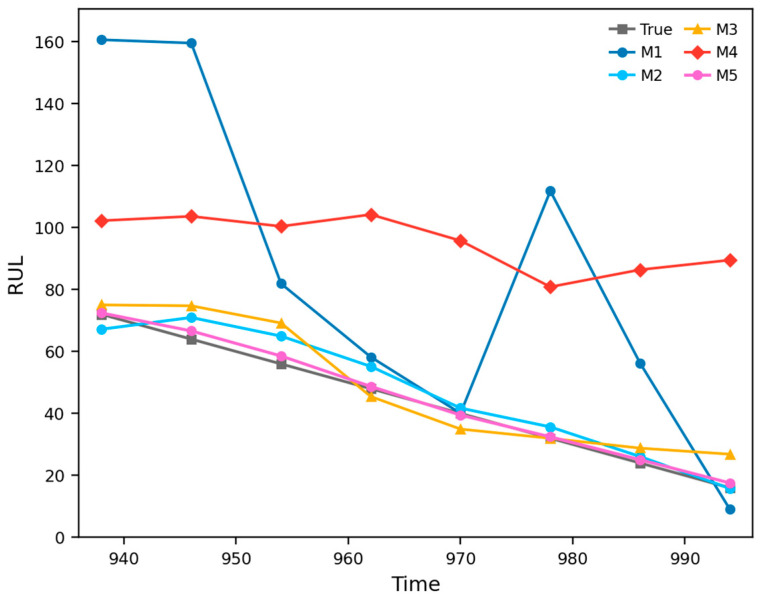
Comparative RUL prediction results on the servo tool holder power head system dataset.

As in Case 1, four methods were selected as benchmark models in Case 2: M1 denotes the power function Wiener process (POW–Wiener) [27], M2 denotes the exponential function Wiener process (EXP–Wiener) [27], M3 denotes the LSTM–Wiener model [28], M4 denotes the power function FBM model (POW-FBM) [25], and M5 denotes the proposed TCN–Transformer–FBM method. The corresponding comparison results are shown in Table 5 and Figure 11.

As shown in Table 5 and Figure 11, the different benchmark methods exhibit substantial performance differences in this case. M1 and M4 show relatively large deviations overall, indicating that when the drift term is constrained to a fixed power-function form, the model cannot adequately adapt to the strongly nonlinear and stage-wise degradation trajectory of the power head system, regardless of whether FBM is introduced. M3 improves upon M1 and M4 to some extent, but its overall accuracy and stability remain insufficient. Among the four benchmark methods, M2 performs the best, suggesting that an exponential-form drift is relatively more suitable for this case; nevertheless, its overall performance still remains clearly inferior to that of M5. By contrast, M5 can more accurately follow the actual RUL evolution trend at all observation points and achieves the best performance in both point prediction accuracy and probabilistic prediction consistency. This indicates that by adaptively learning the drift term through the TCN–Transformer architecture and combining it with FBM-based modeling of the stochastic diffusion process, the proposed method can more effectively characterize the complex temporal evolution and uncertainty dynamics of high-precision mechanical system degradation. Therefore, the advantage of M5 does not arise merely from a stronger network structure, but from a unified probabilistic prediction framework integrating adaptive drift learning and long-memory stochastic diffusion modeling.

## 5. Conclusions

This research proposes a TCN–Transformer–FBM framework for uncertainty-aware remaining useful life prediction of complex mechanical systems. By combining a TCN–Transformer network for adaptive drift learning with an FBM-based stochastic component for diffusion modeling, the proposed method is able to jointly characterize nonlinear degradation trends, long-range temporal dependence, and stochastic uncertainty within a unified prognostic framework. Based on the first hitting time principle, an approximate analytical expression of the RUL probability density function is further derived, which provides a tractable basis for probabilistic prediction and uncertainty quantification. The effectiveness of the proposed framework has been validated on both the XJTU-SY bearing dataset and a real industrial servo tool holder power head system dataset. Experimental results show that the proposed method achieves superior overall performance in terms of both point prediction accuracy and probabilistic prediction quality when compared with the benchmark methods. These findings indicate that the proposed framework can effectively improve the accuracy and robustness of RUL prediction while providing informative uncertainty characterization for maintenance decision-making.

Although the current results are encouraging, broader validation under multiple operating conditions and across more benchmark and industrial datasets is still required to further confirm the robustness and generalizability of the proposed framework. Future work will focus on incorporating additional sources of uncertainty in RUL prediction, particularly by exploring Copula-based methods for multivariate dependence uncertainty modeling when interactions among multiple degradation-related variables need to be explicitly characterized. In addition, the proposed TCN–Transformer–FBM framework will be extended to more degradation scenarios with long-range dependence, and more advanced network architectures will be investigated to further enhance prediction performance.

## Figures and Tables

**Table 1:** Hyperparameters of TCN–Transformer Model.

Hyperparameter Name	Window Length	Num Groups	Num Intervals	Local Finetuned Epochs	Learn Rater
Proposed method	20	24	180	6	5.0000 × 10^−4^

**Table 2:** Performance comparison of different models in the ablation study.

Performance Metrics	RMSE	MAPE(%)	NLL	CRPS
TCN–FBM	12.989	21.009	31.747	9.7002
Transformer–FBM	12.708	20.671	27.808	9.466
Proposed method	8.8448	14.4616	4.3300	6.2024

**Table 3:** Predictive performance comparison of different methods on the XJTU-SY bearing dataset.

Methods	RMSE	MAPE(%)	NLL	CRPS
POW–Wiener [26]	41.179	75.91	4.9002	39.592
EXP–Wiener [26]	20.512	43.962	4.9193	19.241
LSTM–Wiener [28]	47.575	72.905	4.945	43.263
POW-FBM [27]	23.194	39.28	4.9193	26.362
Proposed method	8.8448	14.4616	4.3300	6.2024

**Table 4:** Performance comparison of different models in the ablation study.

Performance Metrics	RMSE	MAPE(%)	NLL	CRPS
TCN-FBM	10.073	27.474	6.0495	8.6125
Transformer–FBM	4.2878	8.4542	4.1124	3.1034
Proposed method	1.513	3.4654	1.8961	0.85832

**Table 5:** Predictive performance comparison of different methods on the servo tool holder power head system dataset.

Methods	RMSE	MAPE(%)	NLL	CRPS
POW–Wiener [26]	137.23	414.4	6.0387	136
EXP–Wiener [26]	5.1739	9.3664	3.1895	3.0647
LSTM–Wiener [28]	137.04	413.54	6.039	135.68
POW-FBM [27]	52.145	164.23	5.5413	34.541
TCN–Transformer–FBM	1.513	3.4654	1.8961	0.85832

## Data Availability

The datasets generated during and/or analysed during the current study are available from the corresponding author on reasonable request.
